# Sex Differences in Intraocular Pressure and Retinal Vessel Responses After Sustained Isometric Knee Extension in Young Adults: A Quasi-Experimental Study

**DOI:** 10.3390/jcm15082858

**Published:** 2026-04-09

**Authors:** Monika Vieversytė-Dvylienė, Vytautas Streckis, Matas Streckis, Rima Solianik

**Affiliations:** 1 Institute of Sport Science and Innovations, Lithuanian Sports University, 44221 Kaunas, Lithuania; monvie@stud.lsu.lt; 2 Department of Coaching Science, Lithuanian Sports University, 44221 Kaunas, Lithuania; vytautas.streckis@lsu.lt (V.S.); matasstreckis@gmail.com (M.S.)

**Keywords:** central retinal arteriolar equivalent, central retinal venular equivalent, IOP, isometric exercise, MVC

## Abstract

**Purpose:** Evidence on the safety of single-joint isometric exercises of the lower legs remains limited. Thus, the aims of this study were to examine intraocular pressure (IOP) and retinal vessel responses during a 1 min isometric knee extension task and to assess potential sex-related differences. **Materials and Methods:** This prospective, parallel-group, quasi-experimental exploratory trial enrolled 43 healthy young adults (22 males and 21 females; age range: 19–35 years), who performed a 1 min sustained maximal-effort isometric knee extension task. The cardiovascular response, IOP, and retinal vessel diameters were assessed. **Results:** The isometric task increased fatigue, heart rate, systolic blood pressure (SBP), and mean arterial pressure in both sexes (*p* < 0.05), with males exhibiting a significantly greater rise in SBP than females (*p* < 0.05). A significant reduction in IOP was observed only in males (*p* < 0.05). Central retinal arteriolar equivalent decreased in both sexes (*p* < 0.05), whereas central retinal venular equivalent (CRVE) increased exclusively in females (*p* < 0.05). Despite the difference in the CRVE response, both sexes exhibited a comparably reduced arteriolar-to-venular diameter ratio (*p* < 0.05). **Conclusions:** To sum up, the isometric maximal-effort knee extension exercise increased cardiovascular loads in both sexes, with males showing a greater SBP rise and a reduction in IOP. Although retinal microvascular responses were sex specific, both sexes showed a similar reduction in the arteriolar-to-venular diameter ratio, indicating a consistent shift in microvascular regulation.

## 1. Introduction

Glaucoma is among the leading causes of irreversible blindness worldwide. Although the vision loss caused by glaucoma cannot be restored, it is largely preventable through early diagnosis and appropriate management [1]. Intraocular pressure (IOP) is the only modifiable risk factor for glaucoma; therefore, its reduction is critical for slowing disease progression [2,3,4]. Emerging evidence indicates that exercise, particularly aerobic activity, and higher levels of physical fitness may offer protection against the development of glaucoma. Furthermore, increased daily physical activity appears to be associated with a slower rate of visual field deterioration in individuals with glaucoma [5].

However, to date, the safety of isometric tasks remains unclear, particularly in relation to lower-extremity exercises. Studies report contradictory findings, with most demonstrating exercise-induced increases in IOP [6,7,8,9,10,11,12] either with [9] or without an effect of individuals’ sex [10]. In contrast, other studies report no significant changes in IOP [13,14,15,16]. Surprisingly, the most extensively investigated tasks are multi-joint isometric resistance exercises such as squatting [6,7,8,10,12,13,14,15], leg press [11], and wall sit [16]. To our knowledge, only one study to date has examined a single-joint isometric exercise—isolated knee flexion and extension performed 20 times for 5 s each—which, unlike multi-joint exercises, was associated with a reduction in IOP [17]. This study included both males and females [17], yet it did not report whether any sex-specific responses were present.

A detailed knowledge of the regulation of retinal blood flow under normal conditions is needed, to understand the pathological alterations of retinal blood flow in retinal diseases [18]. Retinal blood flow is autoregulated, and evidence suggests that the smaller vessels also play a significant role in this regulation [19,20,21,22]. Since fluctuating IOP, especially when changes occur outside the regulatory range, can alter retinal haemodynamics [23], it is essential to consider retinal vessels’ response as a complementary parameter. Surprisingly, to our knowledge, only two studies have examined the effects of lower limb movements, specifically isometric squatting, on retinal vascular responses. Both studies reported retinal artery and vein vasoconstriction in young healthy males [7,14]. However, IOP increased in the study by Jandrasits et al. [7], whereas it remained stable in the study by Lasta et al. [14], suggesting that IOP is unlikely to play a major role in regulating retinal vessel diameter during isometric exercise. It is worth noting that the retinal vascular consequences of localized single-joint lower-limb isometric contractions remain largely uninvestigated, and, to the best of our knowledge, arteriolar and venular responses have not been examined previously.

Single-joint knee strength assessment plays an important role in strength and conditioning, physical therapy, and rehabilitation [24] and in research studies [25,26,27]; therefore, various testing protocols need to be evaluated. It remains unclear whether a sustained contraction would elicit a response similar to that reported by Avunduk et al. [17], specifically a reduction in IOP and whether any sex-specific responses would occur. In view of the gaps identified in the scientific literature, the present study aimed to assess the behaviour of IOP and retinal vessels during a 1 min isometric knee extension task and throughout a 10 min recovery period following exercise cessation, as well as to explore potential differences between males and females. We hypothesized that a 1 min isometric knee extension would reduce IOP and induce retinal vessel vasoconstriction. We also expected to observe sex-related variations in these responses.

## 2. Materials and Methods

### 2.1. Ethical Approval

The present study was approved by The Kaunas Regional Biomedical Research Ethics Committee (No. BE-2-69) and was conducted according to the guidelines laid down in the Declaration of Helsinki. Written informed consent was obtained from all participants during the initial visit. The trial was registered at ClinicalTrials.gov (No. NCT07326462).

### 2.2. Participants

A total of 60 volunteers were assessed for eligibility. The eligibility criteria were as follows: (i) age between 18 and 35 years; (ii) no use of medications or dietary supplements with the potential to influence the experimental variables; (iii) no first-degree family history of glaucoma; (iv) no history of refractive surgery or orthokeratology; (v) no history of systemic or ocular disease; (vi) no history of myopia or hyperopia exceeding ±3.00 D spherical equivalent; and (vii) non-smokers. Females with irregular menstrual cycles or amenorrhoea were not included. Participants were also excluded if they had a history of skeletal or neuromuscular disorders, previous surgery, or any other condition that could impair performance of the isometric task. All participants were recruited through public announcements and social media platforms. The final sample consisted of 43 volunteers of Caucasian ethnicity (22 males and 21 females) who met the inclusion criteria (Table 1).

### 2.3. Experimental Protocol and Study Design

This study employed a quasi-experimental pre–post design to evaluate sex-related differences in IOP and retinal vascular responses to an isometric task, with measurements obtained before the load, immediately after the load, and throughout a 10 min recovery period. Participants were instructed to abstain from moderate to vigorous physical activity for 48 h and to avoid consuming alcohol, caffeine, or other stimulants for 12 h before each testing day. They were also asked to refrain from consuming large meals for 3 h prior to testing and to maintain their habitual hydration practices, ensuring that daily water intake remained within the recommended range (2–3 L/day) while avoiding large fluid intake during the 2 h before exercise to standardize hydration status. Light snacks were permitted up to 2 h before testing. Female participants were not assessed during the menstruation phase. All participants confirmed adherence to these guidelines.

The study took place at the Institute of Sports Science and Innovations, Lithuanian Sports University (Kaunas, Lithuania), from 5 January to 20 February 2026. However, one separate day was allocated for a visit to the “Lirema” eye clinic (Kaunas, Lithuania), during which a comprehensive ocular examination was performed (visual acuity, spherical equivalent, corneal thickness, retinal nerve fibre layer (RNFL) thickness, central macular thickness, lacunarity, tortuosity, and fractal dimension). Day 2 was dedicated to familiarization testing, during which each participant learned to achieve and maintain maximal-effort knee extension, and anthropometric and body composition measurements were obtained. In addition, participants completed the Baecke Questionnaire of habitual physical activity.

**Figure 1:**
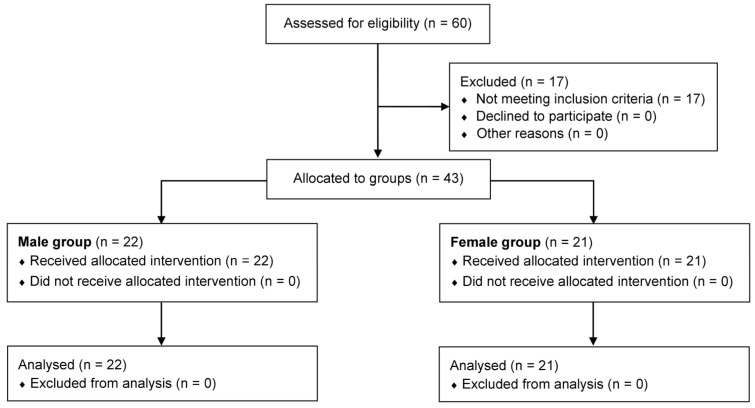
CONSORT flowchart of this study.

All experimental sessions on Day 3 were performed between 16:00 and 18:00 h to control for potential circadian influences on neuromuscular and physical performance [28]. Upon arrival, participants rested in a sitting position for 15 min in a quiet room maintained at an ambient temperature of 23 °C and 67% relative humidity. Retinal photographs were taken, and heart rate (HR), blood pressure (BP), and IOP were then measured. Leg dominance was determined using the Waterloo Footedness Questionnaire-Revised [29]. All participants were right-leg dominant; therefore, the task was performed on the right leg in all cases. After baseline measurements, participants completed a 10 min warm-up on a stationary cycling ergometer (Monark Ergomedic, Monark Exercise AB, Vansbro, Sweden) at moderate intensity, defined as 50–60% of the age-predicted maximal HR or a perceived exertion of 11–12 on the Borg scale [30]. They then performed a 1 min maximal-effort isometric knee extension task, and baseline measurements were repeated immediately after the task, as well as after 5 and 10 min of recovery. No participants were lost, and the data for all 43 participants were available for the analyses (Figure 1).

### 2.4. Anthropometric and Body Composition Measurements

Baseline weight and body fat percentage were measured using a DC-430U body composition analyser (Tanita Corporation, Tokyo, Japan), and height was measured using a Harpenden anthropometer set (Holtain Ltd., Crymych, Wales, UK) while participants wore only underwear and were barefoot. Body mass index (BMI) was calculated as weight divided by height squared.

### 2.5. Habitual Physical Activity Measurement

Habitual physical activity over the previous 12 months was assessed using the Baecke Questionnaire [31]. The questionnaire consists of 16 items organized into three domains of activity: work (8 items), sport (4 items), and leisure (4 items). Items are rated on a 5-point Likert scale ranging from 1 (never) to 5 (always or very often). Each domain can achieve a score in the range of 1 to 5, with higher scores indicating higher levels of habitual physical activity.

### 2.6. Ocular Measurements

Central macular thickness (CMT) and peripapillary RNFL thickness were assessed using a spectral-domain optical coherence tomography (OCT) device (Heidelberg Spectralis OCT, Heidelberg Engineering, Heidelberg, Germany), following the manufacturer’s standard macula and peripapillary scan protocols. CMT was derived from a 6 mm macular raster scan, whereas RNFL thickness was measured using a circular peripapillary scan (3.4 mm diameter). Only high-quality scans that met manufacturer-recommended signal quality criteria were included in the analysis.

Visual acuity was assessed using a Snellen chart, and objective refraction was measured with a Topcon KR-8900 autorefractor (Topcon Medical Systems, Tokyo, Japan). Spherical equivalent was calculated as sphere + ½ cylinder.

### 2.7. Heart Rate, Blood Pressure, and Mean Arterial Pressure Measurements

Heart rate (HR) was measured using a Polar H2 sensor chest strap device (Polar Electro Oy, Kempele, Finland), and blood pressure was measured using an automated blood pressure monitor (Microlife BPA6 PC, Widnau, Switzerland; accuracy ±3 mmHg). Mean arterial pressure (MAP) was calculated from systolic (SBP) and diastolic blood pressure (DBP) values using the standard formula: MAP = DBP + (1/3) × (SBP − DBP) [32].

### 2.8. IOP Measurement

The Icare portable rebound tonometer (Icare, Tiolat Oy, Helsinki, Finland) was used to assess IOP by an experienced ophthalmologist while participants fixated on a target positioned 6 m away. Six consecutive measurements were obtained; the highest and lowest measurements were discarded and the final IOP value was calculated as the mean of the remaining readings. Research has demonstrated that Icare tonometry is a reliable method for screening healthy individuals, with measurements unaffected by factors such as age, sex, axial length, or central corneal thickness [33]. The Icare tonometer has exhibited excellent sensitivity, reaching 98.3% (90–99%, 95% CI), along with an outstanding negative likelihood ratio of 0.024 (0.0088–0.066, 95% CI), making it a valuable tool for ruling out intraocular hypertension [34].

### 2.9. Mean Ocular Perfusion Pressure Measurement

Mean ocular perfusion pressure (MOPP) was calculated from MAP and IOP using the following formula: MOPP = (2/3) × (MAP − IOP) [32].

### 2.10. Cerebrospinal Fluid Pressure Measurement

Cerebrospinal fluid pressure (CSFP) was calculated from BMI and DBP using the following formula: CSFP = 0.44 × BMI + 0.16 × DBP − 0.18 × age − 1.91 [35].

### 2.11. Retinal Vessel Measurement and Analysis

**Figure 2:**
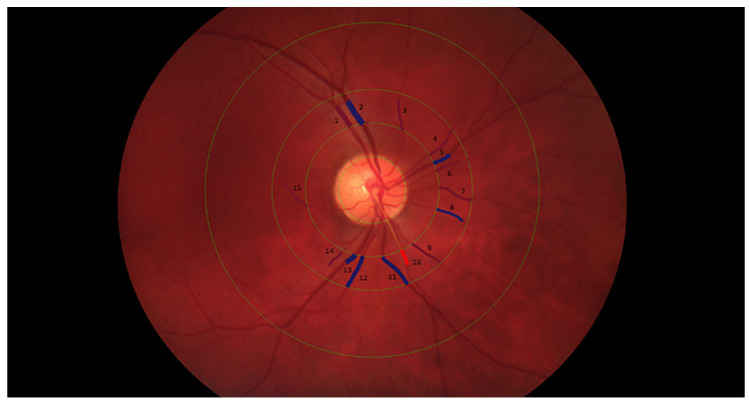
Retinal vessel evaluation with marked measurement zone, highlighting arterioles (1, 3, 4, 6, 7, 9, 10, 14, 15) and venules (2, 5, 8, 11, 12, 13). Note. Image of the right eye fundus obtained in a subject at baseline.

Retinal vessel analysis was previously described by Rusu et al. [36]. Undilated 45° colour optic disc-centred retinal photographs were acquired using an Optomed Aurora handheld non-mydriatic auto-fundus camera (Optomed Plc, Oulu, Finland). This device has been clinically validated for retinal imaging and provides high-quality photographs with strong agreement compared with standard table-top fundus cameras. Previous studies have demonstrated that images obtained with the Optomed Aurora are suitable for quantitative and artificial intelligence (AI)-based retinal analysis, with a high proportion meeting established quality criteria, supporting its use in studies of healthy retinal microvasculature [37,38]. Retinal images then underwent quality assessment based on focus, illumination, contrast, and the absence of artefacts. Following this evaluation, the highest-quality image of the right eye was selected for each participant to ensure reliable measurements. ImageJ version 1.54 (National Institutes of Health, Bethesda, MD, USA) was used to underwent pre-processing for all images using automatic contrast-limited adaptive histogram equalization (CLAHE) to enhance contrast. The pre-processed images were subsequently analysed using the MONA REVA software (version 3.0.0; VITO Health, Mol, Belgium) by two blinded independent graders, following previously published recommendations [39]. Semi–AI-driven retinal analysis using MONA REVA has shown high reliability and reproducibility, supporting its validity for objective retinal microvascular assessment [40,41,42]. For all images, resolution was determined directly from the image data, and the automatically detected optic disc contour was adjusted manually. The optic disc (OD) radius (900 µm) was used for image calibration (µm/pixel) and for defining concentric circles at 2, 3, and 5 radii, with vessels in the 2–3 radius zone automatically segmented and manually refined (Figure 2). Central retinal arteriolar equivalent (CRAE), central retinal venular equivalent (CRVE), and arteriovenous ratio (AVR) were computed by the software using the 6 largest arterioles and venules based on the formula modified by Knudtson et al. [43] or alternatively 4 or 5 vessels when anatomical variation limited vessel identification. The vessel network from 1.5 to 5 OD radii was automatically segmented, skeletonized and then manually refined to correct inaccurate segments.

Retinal vascular network geometry was subsequently quantified using fractal dimension (Df), lacunarity, and tortuosity index automatically calculated by the software. Tortuosity index describes the mean ratio of end-to-end to actual vessel length, lacunarity assesses inhomogeneity of vessel distribution from gaps in the binary image and Df characterizes geometric complexity. Cheung et al. [44] demonstrated that retinal vascular geometry is associated with long-term cardiovascular and metabolic factors rather than acute physiological changes. Consequently, retinal vascular geometric parameters are regarded as stable indicators of microvascular structure. Therefore, only baseline retinal vascular geometry has been reported.

### 2.12. Isometric Task Performance and Maximal Torque Measurement

The 1 min isometric knee extensor task was performed using an isokinetic dynamometer (System 4; Biodex Medical Systems, Shirley, NY, USA). Participants were seated upright in the dynamometer chair with the knee joint positioned at a 120° angle (full extension = 180°). Their arms were crossed over the chest, with hands grasping the trunk-support belt throughout the task. Participants were instructed to produce a maximal voluntary contraction (MVC) of the quadriceps by attempting to extend the knee against the fixed lever arm. Torque was recorded continuously, and data are reported at baseline (3 s) and at the end of the task (59 s). To ensure maximal effort, standardized verbal encouragement was provided by the same experienced researcher. Participants were instructed to maintain a normal breathing pattern throughout the isometric task. Breath-holding was not permitted, as the resulting Valsalva manoeuvre may influence IOP [16,45].

### 2.13. Sample Size Calculation

An a priori power analysis was conducted using GPower software (version 3.1.9.7; Düsseldorf, Germany) based on pilot data from six participants (three males and three females) who completed the study. Using a significance level of α = 0.05 and a statistical power of 0.80, the analysis indicated that a minimum sample size of eight participants was required to detect changes in the primary outcomes: IOP, MOPP, and retinal vessel diameters. However, the public announcement and social networks resulted in a larger number of volunteers than required, all of whom were tested, thereby minimizing the risk of attrition and missing data.

### 2.14. Statistical Analysis

Data are reported as mean ± standard deviation. The normality of the results was assessed using the Shapiro–Wilk test. As the data were non-normal, nonparametric analyses were applied: intervention effects were evaluated with the Wilcoxon signed-rank test, and sex differences were evaluated using the Mann–Whitney U test. The level of significance was set at *p* ≤ 0.05. To determine the effect size (ES) of the exercise-evoked significant changes, ES values were calculated by dividing the Z statistic by the square root of the sample size. Statistical analysis was conducted using IBM SPSS Statistics for Windows (version 29.0.2.0 (20); Armonk, NY, USA) and graphs were created using GraphPad Prism (version 10.4.0 (621); GraphPad Software, San Diego, CA, USA).

## 3. Results

Table 1 presents the baseline characteristics of the participants. As expected from the body composition and anthropometric measures, females had significantly lower body weight (*p* < 0.001) and greater body fat mass percentage (*p* < 0.001). Baseline habitual physical activity and ocular functional and structural parameters did not differ between males and females, except for the RNFL. RNFL values in both groups fell within the normal physiological range of 82–119 μm [46] with females exhibiting higher average RNFL thickness compared with males (*p* = 0.012).

**Figure 3:**
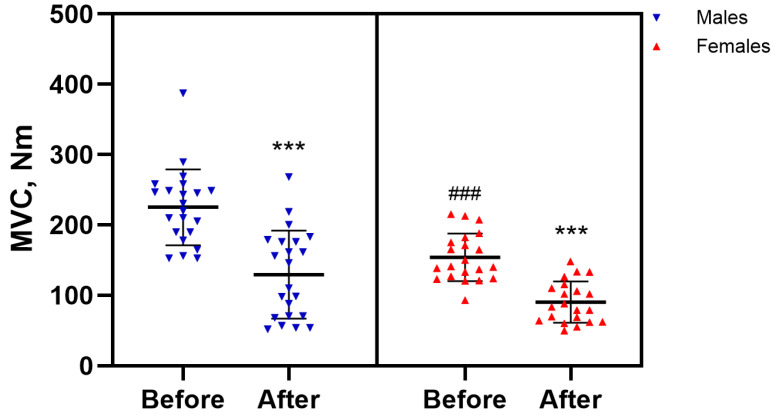
Isometric task–evoked changes in maximal voluntary contraction (MVC) in males and females. Notes. Data are presented as mean (SD). *** *p* < 0.001, compared with baseline; ### *p* < 0.001, compared with males. MVC, maximal voluntary contraction.

As expected, males demonstrated greater baseline strength (*p* < 0.001) (Figure 3). The sustained isometric task significantly reduced maximal quadriceps torque in both males (*p* < 0.001, ES = 0.88) and females (*p* < 0.001, ES = 0.91) (Figure 3). However, the fatigability index did not differ between groups (*p* > 0.05), with values of 43.5 ± 21.0 for males and 40.5 ± 17.0 for females.

**Figure 4:**
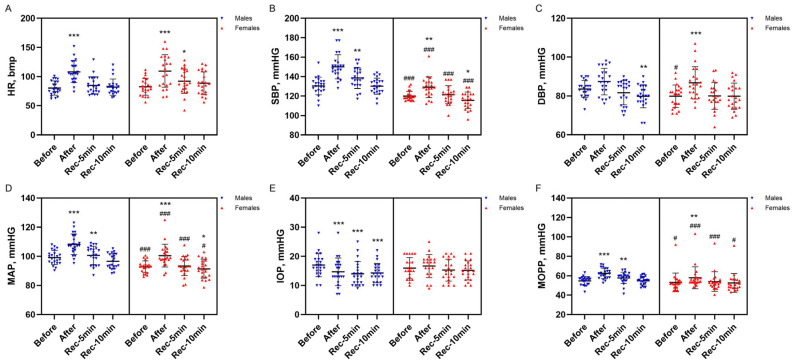
Isometric task–evoked changes in heart rate (HR; (**A**)), systolic blood pressure (SBP; (**B**)), diastolic blood pressure (DBP; (**C**)), mean arterial pressure (MAP; (**D**)), intraocular pressure (IOP; (**E**)), and mean ocular perfusion pressure (MOPP; (**F**)) in males and females. Notes. Data are presented as mean (SD). * *p* < 0.05; ** *p* < 0.01; *** *p* < 0.001, compared with baseline; # *p* < 0.05; ### *p* < 0.001, compared with males.

Figure 4A–D illustrates the systemic hemodynamic responses to the isometric task. As is well documented, significant increases in HR, SBP, and MAP were observed immediately after the isometric task in both the male (*p* < 0.001, ES = 0.88 for HR; *p* < 0.001, ES = 0.87 for SBP; *p* < 0.001, ES = 0.75 for MAP) and female groups (*p* < 0.001, ES = 0.82 for HR; *p* = 0.002, ES = 0.68 for SBP; *p* < 0.001, ES = 0.75 for MAP). A significantly greater increase in SBP was observed in males (12.8 ± 7.2 mmHg) compared with females (6.5 ± 8.1 mmHg) (*p* = 0.009). The task also elicited a significant rise in DBP in the female group (*p* < 0.001, ES = 0.72), whereas only a tendency toward an increase was evident in the male group (*p* = 0.076. ES = 0.38). The elevation in SBP persisted at 5 min of recovery in males (*p* = 0.002, ES = 0.65), while the elevation in HR persisted at 5 min in females (*p* = 0.015, ES = 0.53). By 10 min of recovery, SBP had fallen below baseline in females (*p* = 0.048, ES = 0.43), whereas DBP dropped below baseline in males (*p* = 0.010, ES = 0.55). Meanwhile, HR returned to baseline in both sexes (*p* > 0.05). Across all time points, SBP and MAP remained significantly higher in males compared with females (*p* < 0.001 for SBP; *p* ≤ 0.012 for MAP), whereas DBP was higher in males only at baseline (*p* = 0.010).

Ocular pressure and perfusion responses to the isometric task are presented in Figure 4E,F. A significant decrease in IOP was observed immediately after the isometric task in the male group (*p* < 0.001, ES = 0.69), whereas no significant change was detected in the female group. In males, the reduction in IOP persisted at 5 min (*p* < 0.001, ES = 0.76) and 10 min of recovery (*p* < 0.001, ES = 0.71). No between-group differences in IOP were found (*p* > 0.05). In contrast, MOPP increased significantly immediately after the task in both male (*p* < 0.001, ES = 0.83) and female groups (*p* = 0.003, ES = 0.65). This elevation remained evident at 5 min of recovery only in males (*p* = 0.006, ES = 0.59). Across all time points, MOPP was significantly higher in males than in females (*p* ≤ 0.016). Following the isometric exercise task, CSFP did not change significantly in males (17.6 ± 1.7 mmHg at baseline vs. 18.3 ± 1.8 mmHg after task completion), whereas females exhibited a significant increase from 16.8 ± 2.4 to 17.9 ± 2.6 mmHg (*p* < 0.001, ES = 0.72).

**Figure 5:**
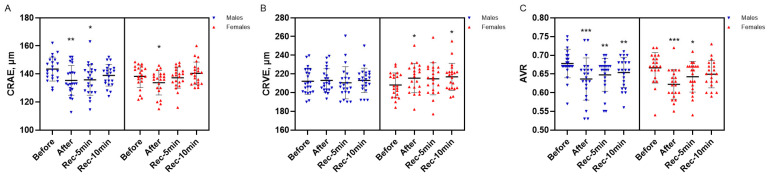
Isometric task–evoked changes in central retinal artery equivalent (CRAE; (**A**)), central retinal vein equivalent (CRVE; (**B**)), and arteriovenous ratio (AVR, (**C**)) in males and females. Notes. Data are presented as mean (SD). * *p* < 0.05; ** *p* ≤ 0.01; *** *p* < 0.001, compared with baseline.

Responses in retinal vessel diameters evoked by the isometric task are presented in Figure 5. A significant vasoconstriction in CRAE was observed immediately after the isometric task in both males (*p* = 0.009, ES = 0.56) and females (*p* = 0.010, ES = 0.57). This vasoconstriction persisted at 5 min of recovery only in the male group (*p* = 0.024, ES = 0.48) (Figure 5A). By contrast, a significant vasodilation in CRVE was observed in the female group immediately after the task and again at 10 min of recovery (*p* = 0.017, ES = 0.52; *p* = 0.011, ES = 0.55, respectively) (Figure 5B). As a consequence, a decrease in AVR was observed immediately after the isometric task in both males (*p* < 0.001, ES = 0.68) and females (*p* < 0.001, ES = 0.72). This reduction persisted at 5 min of recovery in both groups (*p* = 0.004, ES = 0.62 for males; *p* = 0.017, ES = 0.52 for females). After 10 min, a significant decrease remained evident only in the male group (*p* = 0.010, ES = 0.55), whereas in females only a nonsignificant tendency was observed (*p* = 0.080, ES = 0.38) (Figure 5C).

## 4. Discussion

To our knowledge, this study is the first to examine whether sustained isometric knee MVC affects intraocular pressure and retinal vessel diameters in a healthy cohort, and whether these responses differ between sexes. Unique findings of our study are that IOP decreased exclusively in males, even though the isometric task induced elevations in HR, BP, and MAP in both sexes, with greater SBP response in males and greater DBP response in females. The isometric task also led to a sex-specific response regarding the diameter of retinal vessels. Only females exhibited an increase in CSFP, which was accompanied with CRVE vasodilation, while both sexes showed CRAE constriction resulting in a decrease in AVR.

In line with the findings of Solianik et al. [25], we observed similar fatigue in males and females at the end of sustained maximal isometric knee extension. As exercise intensity increases, sympathetic activation contributes progressively to elevations in HR [47]. Consistent with previous findings [7,11], isometric exercise elicited an increase in HR in both males and females. No differences in HR were observed between sexes, with increases of 24.4 ± 12.3 bpm in males and 21.9 ± 17.8 bpm in females. However, the return of HR to baseline following isometric exercise was slower in females, aligning with the findings of Samora et al. [48], who reported a sex-related difference in cardiac vagal reactivation during recovery from isometric handgrip exercise in healthy young adults. Sympathetic activation does not appear to elevate IOP, as the majority of studies show that aerobic exercise, despite substantial increases in HR, consistently leads to a short-term reduction in IOP [49,50].

Noting methodological differences between the present study and earlier investigations is important when interpreting IOP responses. Prior work consistently shows that IOP increases during isometric task execution [6,7,8,9,10,11,12]. However, these elevations are short lived, with IOP typically returning to baseline within approximately 1 min after exercise cessation [10,12]. Findings from Avunduk et al. [17] align with our results, showing an immediate post-exercise IOP decline following repeated isometric knee contractions. This reduction may reflect a compensatory physiological mechanism involving enhanced aqueous humour drainage after acute IOP elevations [10]. Although Avunduk et al. [17] included both sexes, sex-specific responses were not examined. Notably, Vera et al. [10] reported that women exhibit smaller IOP fluctuations between isometric effort and recovery and maintain higher IOP values during recovery. This pattern is consistent with our observation of a post-exercise IOP decline in males but not in females.

As is well established [51,52,53] and as per our results, BP levels are lower in females than males. Moreover, the SBP response after the isometric task was lower in females than in males, and DBP increased only in males. Females rely primarily on increases in cardiac output to raise BP during isometric exercise, whereas males exhibit increases in both cardiac output and total peripheral resistance (TPR) [54]. Because the magnitude of the SBP rise is strongly influenced by TPR, the absence of a TPR-mediated response in females seems to limit the SBP rise and to shift the DBP response pattern. These different patterns resulted in a comparable isometric task-induced increase in MAP in males (8.1 ± 7.5 mmHg) and females (7.1 ± 7.2 mmHg), although males exhibited higher MAP at all time points. This occurs because MAP reflects contributions from both SBP and DBP [32].

Epidemiologic evidence indicates that low ocular perfusion pressure is a significant risk factor for the development and progression of open-angle glaucoma [32,55,56,57]. In the present study, MOPP was consistently lower in females, reflecting their lower MAP across all time points. Despite this baseline difference, the isometric task produced comparable increases in MOPP in males (11.8 ± 8.7 mmHg) and females (7.9 ± 7.7 mmHg) immediately after the isometric task. Although MOPP increased temporarily, this short-term rise should not be interpreted as protective. Higher ocular perfusion pressure is generally associated with a lower risk of glaucoma [32,55,56,57]; however, the increase observed here is transient and therefore unlikely to provide any meaningful long-term benefit. The similar MAP elevations observed in both sexes clearly contributed to these exercise-induced changes in MOPP in both sexes; IOP does not seem to play a meaningful role. These results may also reflect sex-specific haemodynamic balancing strategies that ultimately maintain comparable increases in MOPP.

Microvascular arteriolar function is essential for regulating constant blood flow and sufficient oxygen delivery to maintain organ function [58]. BP-induced vasoconstriction is a key component of vascular autoregulation, ensuring constant blood flow across varying arteriolar BP levels. This mechanism, known as the Bayliss effect, causes smooth muscle cell contraction in response to increased arterial or transmural pressure, resulting in myogenic constriction [59]. Consistent with the BP increases observed in both females and males, and in agreement with previous findings of retinal artery diameter decrease [7,14], an arteriolar (CRAE) vasoconstrictive response was observed. Despite the differences in SBP between sexes, the magnitude of arteriolar vasoconstriction appeared to be sex independent. The sympathetic nervous system (SNS) is a key regulator of vascular function [60], and in the present study, HR used as an indirect marker of SNS activity, did not differ between males and females. Previous studies [7,14] have demonstrated that isometric squatting elicits a time-dependent vasoconstrictive response in retinal veins. It is worth noting that autoregulatory behaviour may differ between measurements obtained during the exercise itself and those taken immediately after its cessation, and the responses of venules may not necessarily parallel those observed in larger retinal veins. Interestingly, a CRVE vasodilative response was observed only in females. Central retinal vein pressure is influenced by CSFP, and larger retinal vein diameters have been associated with elevated CSFP [35]. Accordingly, the greater CSFP estimated in female participants could account for the venular changes observed in females, but not in males. Although retinal microvascular responses were sex specific, both sexes showed a similar reduction in the AVR, indicating a consistent shift in microvascular regulation.

Several limitations should be acknowledged. First, the experimental sample consisted exclusively of healthy, young adults. Therefore, the findings cannot be directly generalized to individuals with glaucoma, those at risk for developing glaucoma, or populations from different age groups. Second, the study assessed only one isometric knee extension task, performed at 100% MVC for 1 min, which may not adequately represent the full spectrum of exercise intensities and durations, and muscle groups, indicating a need for further investigation. More types of isometric exercise should be investigated to identify those that can be safely performed by individuals with glaucoma [16]. Third, findings of exercise-related IOP responses are limited because IOP was not measured during the exercise bout. Prior studies reported transient IOP elevations during exertion [6,7,8,10,12,13,14,15] and showed that these increases dissipate rapidly once exercise stops [10,12]. However, participants tended to forcefully clench their eyes during the isometric maximal effort knee extension, and we were unable to measure IOP during task performance. Because these IOP spikes are short-lived, it is possible that forced eyelid closure during maximal effort could have masked momentary IOP increases. In addition, even eyelid squeezing has been shown to transiently increase IOP by 3.8 ± 0.6 mmHg [61]. Thus, our data primarily reflect ocular responses after an isometric task rather than changes occurring during the task, when IOP may have already declined; therefore, the direct applicability of our findings to ocular health is limited.

## 5. Conclusions

The findings indicate that a 1 min isometric maximal-effort knee extension increased the cardiovascular load in both sexes, with males showing a greater SBP rise and a reduction in IOP. Although retinal microvascular responses were sex-specific, both sexes showed a similar reduction in the arteriolar-to-venular diameter ratio, indicating a consistent shift in microvascular regulation. Our findings demonstrate that although the level of fatigue evoked during the isometric task did not differ between participants, the physiological responses were sex-specific. As our measurements were obtained immediately after, rather than during, the exercise bout, the interpretation of these responses in the context of glaucoma risk should be approached with caution. Nevertheless, these findings further highlight the need for individualized interpretation of exercise-induced physiological outcomes and support the consideration of sex-specific strategies affecting IOP in future research.

## Figures and Tables

**Table 1:** Baseline characteristics of the participants.

	Male Group (n = 22)	Female Group (n = 21)
Age, years	27.6 (5.3)	26.2 (5.1)
Weight, kg	86.6 (13.9)	67.7 (11.3) ###
Body mass index, kg/m^2^	24.8 (3.4)	24.2 (4.3)
Body fat, %	14.7 (6.3)	26.9 (9.8) ###
Physical activity	10.2 (2.50)	10.0 (2.53)
Work	2.85 (0.88)	2.74 (0.93)
Sports	3.77 (1.32)	3.67 (1.29)
Leisure	3.57 (1.06)	3.61 (1.05)
Visual acuity	0.87 (0.24)	1.19 (1.91)
Spherical equivalent, D	−0.36 (1.06)	−0.75 (0.72)
Retinal nerve fiber layer, µm	97.9 (3.9)	102.3 (6.0) #
Corneal thickness, µm	544.8 (19.7)	548.3 (31.2)
Central macular thickness, µm	258.1 (10.3)	258.3 (11.0)
Lacunarity	1.12 (0.02)	1.11 (0.03)
Tortuosity	0.87 (0.01)	0.87 (0.01)
Fractal Dimension	1.46 (0.03)	1.44 (0.04)

Notes. Data are presented as mean (SD). # *p* < 0.05; ### *p* < 0.001, compared with males.

## Data Availability

The datasets generated and/or analyzed during the current study are not publicly available because participants were not informed of this option and their consent was not obtained; however, they are available from the corresponding author upon reasonable request.
